# Clonal Expansion and Emergence of Environmental Multiple-Triazole-Resistant *Aspergillus fumigatus* Strains Carrying the TR_34_/L98H Mutations in the *cyp*51A Gene in India

**DOI:** 10.1371/journal.pone.0052871

**Published:** 2012-12-28

**Authors:** Anuradha Chowdhary, Shallu Kathuria, Jianping Xu, Cheshta Sharma, Gandhi Sundar, Pradeep Kumar Singh, Shailendra N. Gaur, Ferry Hagen, Corné H. Klaassen, Jacques F. Meis

**Affiliations:** 1 Department of Medical Mycology, Vallabhbhai Patel Chest Institute, University of Delhi, Delhi, India; 2 Department of Biology, McMaster University, Hamilton, Ontario, Canada; 3 Department of Pulmonary Medicine, Vallabhbhai Patel Chest Institute, University of Delhi, Delhi, India; 4 Department of Medical Microbiology and Infectious Diseases, Canisius Wilhelmina Hospital, Nijmegen, The Netherlands; 5 Department of Medical Microbiology, Radboud University Nijmegen Medical Centre, Nijmegen, The Netherlands; Instituto de Salud Carlos III, Spain

## Abstract

Azole resistance is an emerging problem in *Aspergillus* which impacts the management of aspergillosis. Here in we report the emergence and clonal spread of resistance to triazoles in environmental *Aspergillus fumigatus* isolates in India. A total of 44 (7%) *A. fumigatus* isolates from 24 environmental samples were found to be triazole resistant. The isolation rate of resistant *A. fumigatus* was highest (33%) from soil of tea gardens followed by soil from flower pots of the hospital garden (20%), soil beneath cotton trees (20%), rice paddy fields (12.3%), air samples of hospital wards (7.6%) and from soil admixed with bird droppings (3.8%). These strains showed cross-resistance to voriconazole, posaconazole, itraconazole and to six triazole fungicides used extensively in agriculture. Our analyses identified that all triazole-resistant strains from India shared the same TR_34_/L98H mutation in the *cyp*51 gene. In contrast to the genetic uniformity of azole-resistant strains the azole-susceptible isolates from patients and environments in India were genetically very diverse. All nine loci were highly polymorphic in populations of azole-susceptible isolates from both clinical and environmental samples. Furthermore, all Indian environmental and clinical azole resistant isolates shared the same multilocus microsatellite genotype not found in any other analyzed samples, either from within India or from the Netherlands, France, Germany or China. Our population genetic analyses suggest that the Indian azole-resistant *A. fumigatus* genotype was likely an extremely adaptive recombinant progeny derived from a cross between an azole-resistant strain migrated from outside of India and a native azole-susceptible strain from within India, followed by mutation and then rapid dispersal through many parts of India. Our results are consistent with the hypothesis that exposure of *A. fumigatus* to azole fungicides in the environment causes cross-resistance to medical triazoles. The study emphasises the need of continued surveillance of resistance in environmental and clinical *A. fumigatus* strains.

## Introduction

*Aspergillus fumigatus* is the commonest etiologic agent of various clinical forms of bronchopulmonary aspergillosis including allergic, acute invasive and chronic pulmonary aspergillosis (CPA). The disease has a global distribution and it is widespread in India [1]. Invasive aspergillosis is the most severe manifestation with an overall annual incidence varying from 2 to 10% in the immunosuppressed patient population whereas CPA affects primarily immunocompetent individuals with an estimated prevalence of 3 million worldwide [2], [3]. Azoles, such as itraconazole, voriconazole, and posaconazole are among the recommended first-line drugs in the treatment and prophylaxis of aspergillosis [4], [5]. Azole resistance is an emerging problem in *A. fumigatus* in Europe and has been shown to be associated with increased probability of treatment failure [6]–[8]. Azole resistance is commonly due to mutations in the *cyp*51A gene, which encodes 14-α-demethylase in the ergosterol biosynthesis pathway. In azole-resistant clinical *A. fumigatus* isolates a wide variety of mutations in the *cyp*51A gene have been found, such as substitutions at codons G54, G138, P216, F219, M220 and G448 [9]–[12]. However, in the Netherlands a different resistance mechanism consisting of the L98H substitution, together with a 34-bp tandem repeat (TR_34_) in the promoter region of this gene (TR_34_/L98H) was found to be present in over 90% of azole resistant isolates [13]. The TR_34_/L98H resistance mechanism has been endemic in the Netherlands and subsequently reported from other European countries such as Denmark, France, Germany, Spain and the United Kingdom [12], [14]–[19].

Isolates of *A. fumigatus* with TR_34_/L98H mutations exhibit a pan-azole resistant phenotype and were recovered primarily from azole-naive patients and from environmental sources in the Netherlands and Denmark [15], [17], [20], [21]. These observations suggest that patients acquire azole-resistant *Aspergillus* from environmental sources rather than arising through azole therapy. The consequence of this type of resistance development is that patients at risk can be exposed to and infected by azole-resistant strains in the environment. Furthermore, TR_34_/L98H isolates were cross-resistant to certain azole fungicides employed extensively in agriculture for crop protection against phytopathogenic molds, to prevent post-harvest spoilage [21]. An environmental route of resistance development poses a major challenge because multiplication and spread of resistant strains in the environment can be anticipated. Recently, we reported from India the occurrence of TR_34_/L98H mutations in the c*yp*51A gene in *A. fumigatus* isolates from patients with chronic respiratory disease who had not previously been exposed to azoles [22]. This emergence of resistance in Indian clinical isolates prompted us to undertake a wide environmental survey of azole resistant *A. fumigatus* isolates in India. Herein, we report multi-triazole resistant environmental *A. fumigatus* isolates from India harboring TR_34_/L98H mutations in the c*yp*51A gene, from soil samples of paddy fields, tea gardens, cotton trees, flower pots and indoor air of hospital. Furthermore, we investigated the cross resistance of these environmental and clinical TR_34_/L98H *A. fumigatus* isolates to registered and commonly used azole fungicides in India and determined the genetic relatedness of Indian environmental and clinical *A. fumigatus* isolates harboring the TR_34_/L98H mutations and compared them with isolates from Europe and China.

## Results

### Isolation of Environmental Strains of *A. fumigatus*

**Figure 1:**
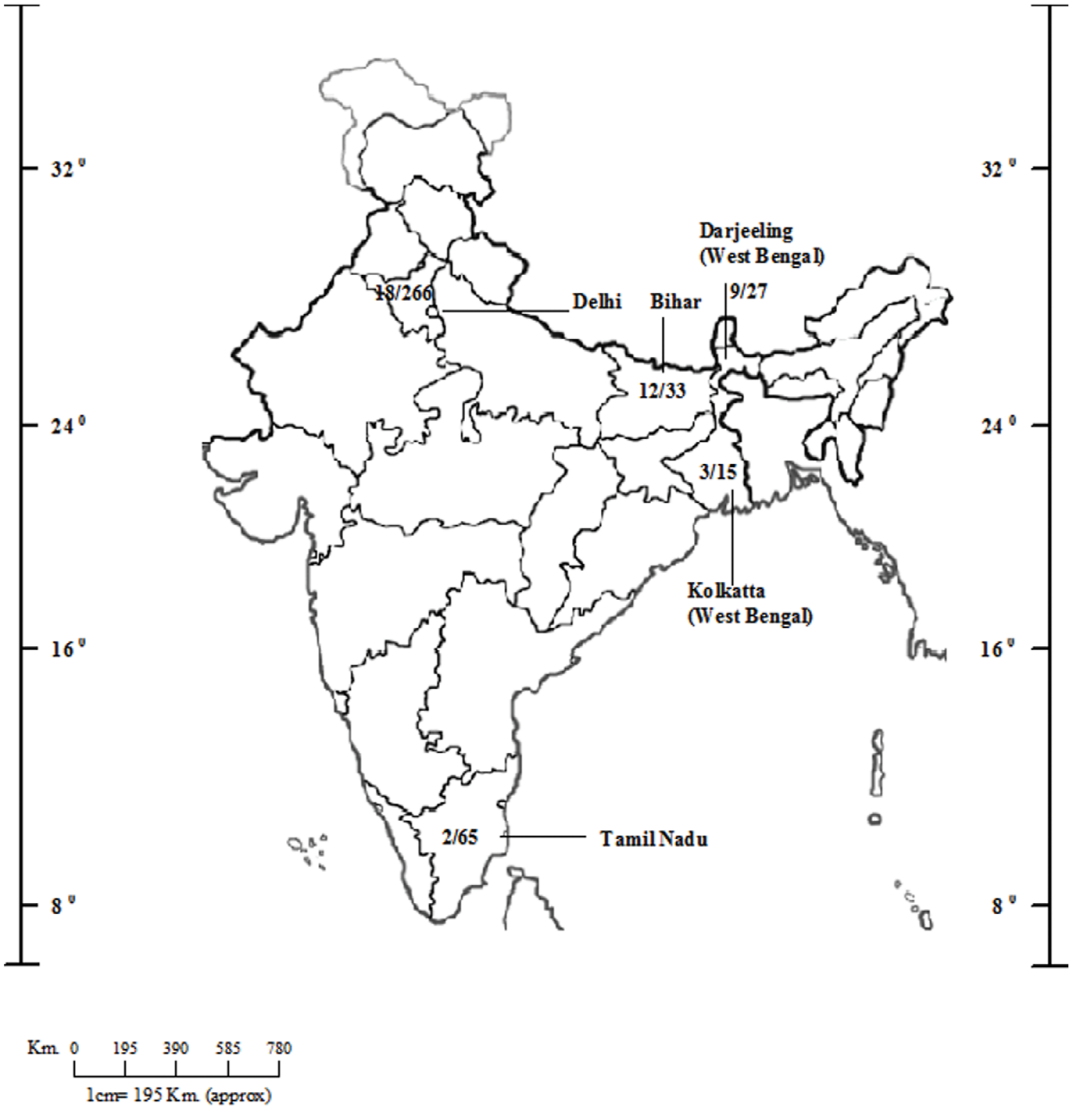
An outline map of India showing state-wise isolation of multiple-triazole resistant *Aspergillus fumigatus* isolates from variety of environmental samples.

Of the 486 environmental samples tested, 201 (41.4%) showed the presence of *A. fumigatus* in all types of substrates tested except nursery plants soil and decayed wood inside tree trunk hollows. The data of state-wise distribution and prevalence of azole resistant *A. fumigatus* in soil and air samples is presented in Table 1 and Figure 1. Of the 201 *A. fumigatus* positive samples, 630 individual *A. fumigatus* colonies were obtained from Sabourauds dextrose agar (SDA) plates. The count of *A. fumigatus* on primary SDA plate ranged from one colony to confluent growth. Besides *A. niger, A. flavus*, *A. terreus*, other molds such as mucorales, and *Penicillium* species were also observed in soil samples. Out of 630 *A. fumigatus* colonies tested, 44 (7%) isolates originating from 24 samples grew on SDA plates containing 4 mg/L itraconazole. Among these 44 itraconazole-resistant (ITC+) isolates, 15 were obtained from different potted plants of the V. P. Chest Institute (VPCI) garden, Delhi, 12 from rice paddy fields in Bihar, 9 from tea gardens in Darjeeling, 3 each from soil beneath cotton trees (*Bombax ceiba*) from Kolkata and from aerial sampling of patient rooms of the VPCI hospital, and 2 from soil containing bird droppings in Tamil Nadu (Table 1). Overall, 5% (24/486) of the samples tested harbored itraconazole resistant *A. fumigatus*. Among the positive samples, 11.9% (24/201) showed at least one colony of resistant *A. fumigatus*. The isolation rate of itraconazole resistant *A. fumigatus* was highest 33% (9/27) from the soil of tea gardens followed by soil from flower pots of the hospital garden 20% (15/75), soil beneath cotton trees 20% (3/15), rice paddy fields 12.3% (12/97), air samples of hospital wards 7.6% (3/39) and from soil admixed with bird droppings 3.8% (2/52). There was no isolation of resistant *A. fumigatus* isolates from soil samples of public parks and gardens inside the hospital premises and red chilly fields in Tamil Nadu.

**Table 1:** State-wise distribution of environmental *Aspergillus fumigatus* isolates with TR_34_/L98H mutations from India.

	No. of *A. fumigatus* isolates with TR_34_/L98H mutations/No. of isolates tested n = 44/630 (201/486)*									
	Garden soil	Paddy/Rice/Red chilly fields soil	Tea garden soil	Tree trunk hollow wood	Aerial isolations from hospital wards	Nursery flower pots soil	Soil beneath cotton trees	Garden soil of hospitals	Flower pots soil of hospital garden	Soil with bird droppings
**UT** **of Delhi (n = 266)**										
VPCI, DU	–	–	–	–	3/7 (14/39)	–	–	0/20 (10/10)	15/120 (30/75)	–
Ashok Vihar Park	0/0(0/10)	–	–	–	–	–	–	–	–	–
Lodhi garden	0/80(20/45)	–	–	–	–	–	–	–	–	–
Central Park, DU	0/27(10/50)	–	–	–	–	–	–	–	–	–
Police Lines, DU	–	–	–	0/0(0/12)	–	–	–	–	–	–
Gulabi Bagh	–	–	–	–	–	0/0(0/25)	-	–	–	–
**Tamil Nadu (n = 65)**Thorapadi Village	–	0/4(2/13)	-	–	–	–	–	–	–	–
Kanchipuram	–	–	–	–	–	–	–	–	–	2/25 (19/52)
**West Bengal (n = 59)**Hoogli Dist., Kolkata	–	–	–	–	–	–	3/5(1/15)	–	–	–
Siliguri	–	0/40(13/17)	-	–	–	–	–	–	–	–
Darjeeling	–	-	9/51(16/27)	–	–	–	–	–	–	–
**Bihar (n = 33)** Munger	–	12/78(26/33)	–	–	–	–	–	–	–	–
**Uttrakhand (n = 21)** Kedar, Basora	–	0/108(21/21)	–	–	–	–	–	–	–	–
**Haryana (n = 21)** Jajjhar	–	0/60(15/21)	–	–	–	–	–	–	–	–
**Meghalaya (n = 11)**Shillong	–	0/0(0/5)	–	–	–	–	–	0/0(0/6)	–	–
**Sikkim (n = 6)** Gangtok	0/5(4/6)	–	–	–	–	–	–	–	–	–
**Himachal Pradesh (n = 4)** Dalhousie	0/0(0/4)	–	–	–	–	–	–	–	–	–

* Parenthesis denotes the numerator as number of samples positive for *A. fumigatus,* denominator denotes the number of samples tested; ^†^UT, Union Territory; VPCI, V. P. Chest Institute; DU, Delhi University.

### Evidence for Cross-Resistance to Triazole Antifungal Drugs

All the 44 ITC+ *A. fumigatus* isolates from the environment showed reduced susceptibility to azoles. The geometric mean (GM) MIC of itraconazole (GM, 16 mg/L) was the highest, followed by voriconazole (GM, 8.7 mg/L), and posaconazole (GM, 1.03 mg/L). All the antifungal drugs tested showed reduced efficacy against all the ITC+ *A. fumigatus* isolates (Table 2), consistent with cross-resistance of these isolates to the tested azoles. Among the triazoles, the MIC difference between wild type and TR_34_/L98H isolates were the highest for itraconazole (r = 0.96) followed by voriconazole (r = 0.91) and posaconazole (r = 0.72). Of the10 fungicides, 7 showed dissimilarity between the MICs with greatest differences found for bromuconazole, difenoconazole, tebuconazole (r = 0.96 each) followed by hexaconazole (r = 0.95), epoxiconazole (r = 0.92), metconazole (r = 0.89) and lowest for cyproconazole (r = 0.22) (Table 2).

**Table 2:** In- vitro antifungal susceptibility profile of medical triazoles and triazole fungicides against environmental and clinical Aspergillus fumigatus isolated in India.

	MIC* (mg/L)												
Triazoledrugs andfungicides	Environment						Clinical						Effect size r
	TR_34_/L98H (n = 44)			251676672Wild type (n = 22)			TR_34_/L98H (n = 9)			Wild type (n = 13)			
	GM*	MIC_50_ *	Range	GM	MIC_50_	Range	GM	MIC_50_	Range	GM	MIC_50_	Range	
Itraconazole	16	16	16–>16	0.43	0.5	0.25–1	16	16	16>16	0.11	0.125	0.03–1	0.96
Voriconazole	8.7	8	4–16	0.65	0.5	0.25–1	5.9	8	2–16	0.10	0.125	0.03–0.25	0.91
Posaconazole	1.03	1	0.5–2	0.46	0.5	0.06–1	3.2	2	1–>8	0.25	0.25	0.125–1	0.72
Bromuconazole	31.4	32	16–>32	2.5	2	1–4	32	32	32–>32	2.2	2	1–4	0.96
Cyproconazole	32	32	32–>32	30.9	32	16–>32	32	32	32–>32	29.4	32	16–32	0.22
Difenoconazole	31.4	32	16–>32	2.0	2	1–8	32	32	32–>32	1.8	2	0.5–4	0.96
Epoxiconazole	32	32	32–>32	5.2	4	2–16	32	32	32–>32	4.1	4	2–8	0.92
Hexaconazole	31	32	8–>32	4.87	4	2–8	32	32	>32	3.39	4	2–8	0.95
Metconazole	3.8	4	1–8	0.3	0.5	0.125–1	4	4	2–16	0.4	0.5	0.25–2	0.89
Penconazole	32	32	32–>32	30.9	32	32–>32	32	32	32–>32	32	32	32–>32	0
Tebuconazole	31.4	32	16–>32	2.6	2	1–8	32	32	>32	3.0	4	1–8	0.96
Triadimefon	32	32	>32	32	32	>32	32	32	>32	32	32	32–>32	0
Tricyclazole	32	32	32–>32	32	32	32–>32	32	32	>32	32	32	32–>32	0

* Minimum inhibitory concentration; GM, geometric mean.

### Evidence for Clonal Spread of a Single Triazole-Resistant *A. fumigatus* Genotype

**Figure 2:**
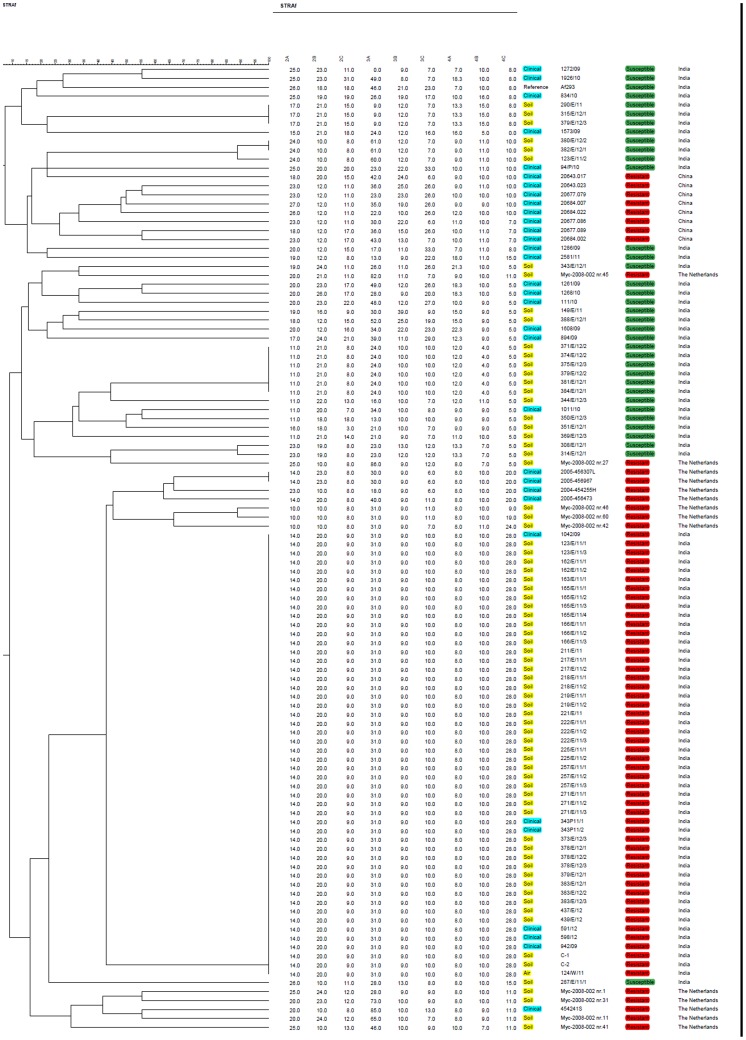
Genotypic relationship between the wild-type and TR_34_/L98H *Aspergillus fumigatus* (clinical and environmental isolates from India, The Netherlands and France) and TR_34_/L98H *A. fumigatus* (clinical isolates from China and Germany). The dendrogram is based on a categorical analysis of 9 microsatellite markers in combination with UPGMA clustering. The scale bar indicates the percentage identity. Clinical: blue, Environmental: yellow, Resistant: red, Susceptible: green.

Our genotype analyses identified that all of the 44 ITC+ *A. fumigatus* isolates from India exhibited the same TR_34_/L98H genotype at the *cyp*51A gene. Furthermore, these strains had the same allele across all nine examined microsatellite loci (Fig. 2). In contrast to the genetic uniformity of azole-resistant strains from India, the azole-susceptible isolates from both patients and environments in India were genetically very diverse. Indeed, all nine loci were highly polymorphic in populations of azole-susceptible isolates from both clinical and environmental samples.

### Origin(s) of the Azole-resistant *A. fumigatus* Genotype in India

**Figure 3:**
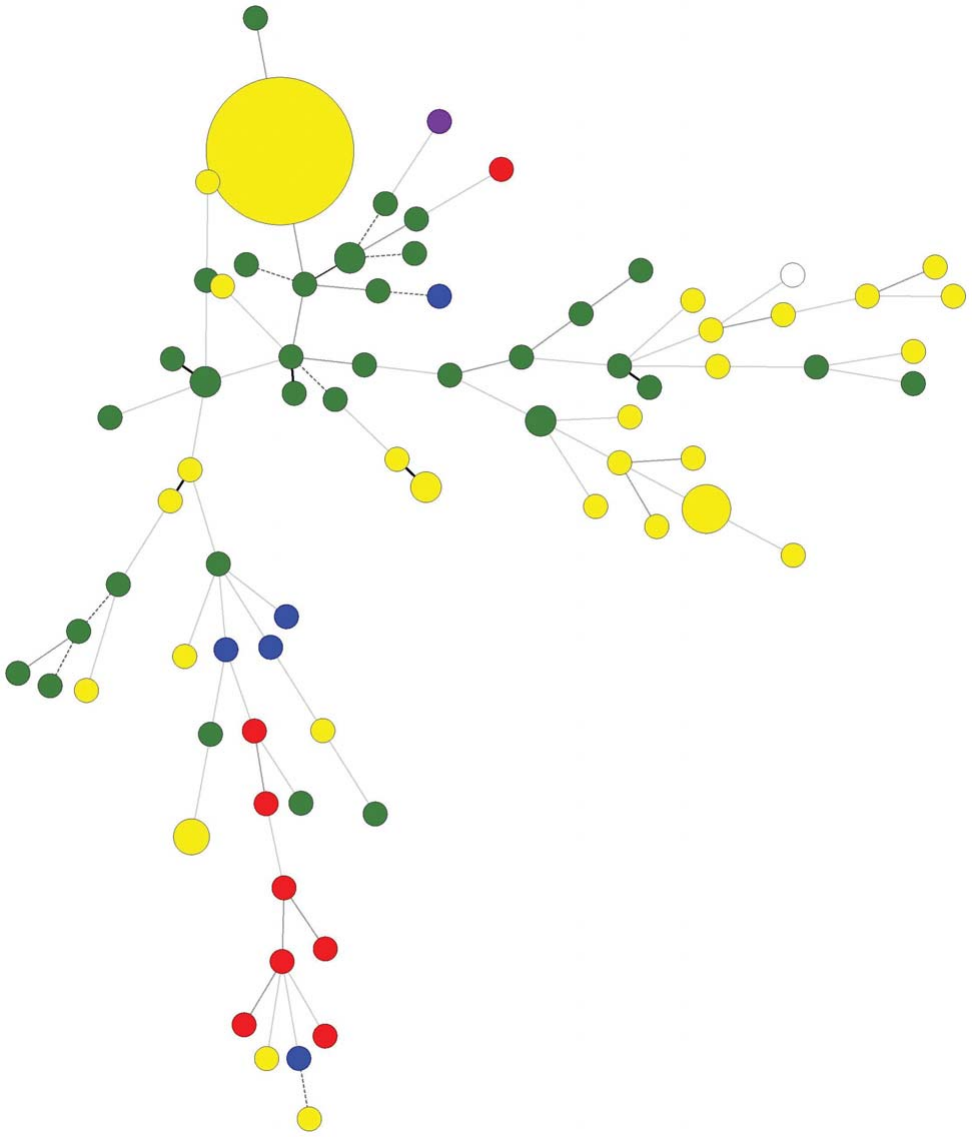
Minimum spanning tree showing wide genotypic diversity in the TR_34_/L98H and wild type *A. fumigatus* isolates studied. The figure shows the 74 different genotypes (circles), the number of strains belonging to the same genotype (sizes of the circles), and origin of isolates (circles in yellow indicate Indian isolates; green Dutch isolates; red Chinese isolates; blue French isolates, purple German isolate and white reference strain, AF293). Solid thick and thin branches indicates 1 or 2 microsatellite markers differences, respectively; dashed branches indicates 3 microsatellite markers difference between two genotypes; 4 or more microsatellite markers differences between genotypes are indicated with dotted branches.

The widespread occurrence of a single azole-resistant genotype across India contrasts with those found in several other regions outside of India. In our analyses, a diversity of genotypes has been found for clinical TR_34_/L98H azole-resistant *A. fumigatus* strains in China, France, Germany and in both clinical and environmental sources in the Netherlands (Figs. 2 and 3). To examine the origin(s) of the azole - resistant genotype in India, we first attempted to isolate azole - susceptible strains from the 24 soil samples that contained the 44 azole-resistant strains. Among these 24 soil samples, we successfully obtained and analyzed eight azole-susceptible isolates from seven of the 24 samples through dilution plating, single colony purification, and screening using itraconazole-containing and non-containing media. Our genotype analyses using the 9 microsatellite markers revealed that none of the eight strains had a genotype identical to the azole-resistant genotype in India. These eight azole-susceptible strains belonged to four different genotypes. Interestingly, three of the genotypes shared no allele with the azole-resistant genotype at any of the nine microsatellite loci while the remaining genotype shared an allele with the azole-resistant genotype at only one of the nine loci.

To further explore the potential origin(s) of the azole-resistant genotype in India, we further analyzed the genotypes of all the azole-susceptible strains from within India. Among the nine microsatellite loci, we were able to find allele-sharing at only six loci between the Indian azole-resistant genotype and the 35 azole-susceptible clinical and soil/air isolates in India. The highest number of loci with shared alleles between any of the 35 azole susceptible strains and the resistant genotype was at only two of the nine loci. Therefore, even with free recombination among the genotypes represented by the 35 azole susceptible strains in India, the azole-resistant genotype could not be generated due to the lack of corresponding alleles at three of the nine loci (loci 2A, 3A, and 4C, Fig. 2) found only in the azole-resistant strains.

Interestingly, though not identical, several strains from outside of India were found to have genotypes more similar to the Indian azole-resistant strains than the Indian azole-susceptible strains (Fig. 2). For example, ten of the 51 strains from outside of India shared alleles in at least four of the nine loci with the Indian azole –resistant genotype, with four of the 10 strains sharing alleles at five loci. These 10 strains were all similarly resistant to azoles as the Indian azole-resistant genotype and all 10 strains carried the same TR_34_/L98H mutation. The combined allelic comparisons identified that the azole-resistant strains from outside of India contained alleles at seven of the nine microsatellite loci found in the Indian azole-resistant genotype, one more than all the Indian azole-susceptible strains combined. At locus 2A, only the sample from outside India contained the allele #14 (in 8 of the 51 strains) found in the Indian azole-resistant genotype while the azole-susceptible sample from India did not contain this allele (Fig. 2). However, a reverse situation occurred at locus 2C where allele #9 in the Indian azole-resistant genotype was found in the Indian azole-susceptible population (in one of the 35 strains) but not from outside of India. Finally, different from the other eight loci, locus 4C had a unique allele (#28) found only in the Indian azole-resistant strains and this allele was absent from any other strains in the whole analyzed sample, either from within or outside of India (Fig. 2).

## Discussion

The site specific mode of action and intensive use of demethylase inhibitors (DMIs) fungicides for post harvest spoilage crop protection against phytopathogenic molds, has led to the development of resistance in many fungi of agricultural importance. It is anticipated that the excessive use of azoles in agriculture would not only influence the plant pathogenic fungi but also would inevitably influence susceptible species of the saprophytic flora [23]. Many potentially human pathogenic fungi such as *Coccidioides*, *Histoplasma*, *Aspergillus*, and *Cryptococcus* have their natural habitats in the environment and in many instances the infecting fungal organisms are acquired from the surrounding environment. Recently the use of azole-based agricultural chemicals has also been implicated as a major factor in the increase in frequency of multiple-triazole-resistant (MTR) isolates of *A. fumigatus* infecting humans by selection of MTR alleles [24], [25]. This is supported by a recent report originating from the Netherlands that showed over 90% of Dutch azole resistant *A. fumigatus* isolates recovered from epidemiologically unrelated patients clustered onto a single lineage [13]. In the present study 7% of the Indian environmental *A. fumigatus* isolates were multi-triazole resistant with a single resistant mechanism carrying the TR_34_/L98H mutation in the c*yp*51A gene (Table 1). The resistant isolates were recovered from soil samples of potted plants, paddy fields and tea gardens where certain triazole fungicides (tebuconazole, hexaconazole, and epoxiconazole) were extensively used. Although, Europe leads the world in usage of agricultural fungicides (40%) followed by Japan and Latin America, in India usage of fungicides is increasing and current fungicide use in India is 19% of the total pesticide use [26]. In the USA the use of azoles in agriculture is insignificant as compared to Europe (http://ec.europa.eu/food/fs/sc/ssc/out278_en.pdf). Consequently, there has been no report of finding the TR_34_/L98H mutation in clinical or environmental isolates in the USA. But this resistance type has been found in the environment in Europe and now also in India. It is noteworthy that so far no environmental survey of TR_34_/L98H *A. fumigatus* isolates outside Europe has been reported. The fungicides belonging to different chemical groups have been registered in India only in the past two decades and these are being used against diverse diseases in fruits, vegetables, plantation crops and some field crops [26]. Triazole fungicides such as hexaconazole, propiconazole, triadimefon, and tricyclazole account for a substantial fungicide market in India [26]. Overall, the highest fungicide usage in India is on pome fruits (12.7%), followed by potatoes (12.2%), rice (12%), tea (9.4%), coffee, chillies, grapevines, other fruits and vegetables [26]. Also, triazole fungicides are characterized by their long persistence in soil. Singh and Dureja demonstrated that hexaconazole persist longer in Indian soil due to its hydrophobic nature [27]. In India, the maximum amounts of fungicide usage are found in southern India, followed by western, eastern and northern Indian states. In this study the multi triazole resistant *A. fumigatus* carrying the TR_34_/L98H genotype was isolated from Union Territory (UT) of Delhi (northern region), West Bengal and Bihar (eastern region of India about 1100 Km from the North) and Tamil Nadu (southern region of India, about 2100 Km from the North) states. The western region of India has yet to be surveyed but considering the high usage of fungicides in this region, isolation of azole resistant *A. fumigatus* may be anticipated.

Previous environmental surveys of azole resistant *A. fumigatus* have only been reported from Europe (the Netherlands and Denmark) and those surveys identified that 12% (6/49) of Dutch soil samples and 8% (4/50) of Danish soil samples were positive for the TR_34_/L98H genotype [15], [17]. Only one other mutation in the *cyp*51A gene combined with a different tandem repeat (TR_46_/Y121F/T289A) that was putatively linked to an environmental origin has been reported from clinical samples [28] and this genotype constituted 36% of resistant isolates in a Dutch referral centre [29]. The present study represents one of the largest environmental surveys of multi-triazole resistant *A. fumigatus* done so far and detected that 7% of the *A. fumigatus* isolates and 5% of soil/aerial samples distributed across large areas of India carried one single resistant mechanism. Culture of soil samples taken from potted plants (where commercial compost was used) and kept inside the hospital premises were positive for the same genotype. In contrast, natural soil sampled from the gardens of Delhi and hospitals did not grow the resistant *A. fumigatus* isolates although they were positive for *A. fumigatus*. Our findings corroborate with the findings of a Dutch environmental report where none of the *A. fumigatus* isolates obtained from natural soil was found to be azole resistant [15]. Therefore, environmental surveys for detection of genotype TR_34_/L98H resistant *A. fumigatus* isolates may focus on sampling of soil from fields and commercial compost where fungicides are invariably used. It is noteworthy that the air samples of patient’s wards of VPCI hospital harboured the same genotype of multi-triazole resistant *A. fumigatus*, isolated on two different occasions which raises concern on the exposure of hospitalized patients to this resistant genotype. In this context it is pertinent to mention that previously multi-triazole resistant TR_34_/L98H *A. fumigatus* isolates have been reported from patients attending the outpatient departments of VPCI who were never exposed to azoles [22]. In addition multi-triazole resistant *A. fumigatus* has also been isolated from admitted patients of VPCI. The presence of *A. fumigatus* resistant to medical triazoles poses a threat to immunocompromised patients as alternative therapy is limited.

Snelders et al. reported that TR_34_/L98H isolates from clinical and environmental origins were cross resistant to five triazole DMIs fungicides, propiconazole, bromuconazole, tebuconazole, epoxiconazole and difenoconazole and thus supporting the hypothesis that exposure of *A. fumigatus* to azole fungicides in the environment causes cross resistance to medical triazoles. [21]. Furthermore, these investigators also reported that these five triazole DMIs showed very similar molecule structures to the medical triazoles and adopted a similar conformation while docking the target enzyme and exhibit activity against wild type *A. fumigatus* but not against multi-triazole resistant TR_34_/L98H *A. fumigatus* [21]. Similarly, in the present study four of the five (bromuconazole, tebuconazole, epoxiconazole and difenoconazole) triazole DMIs known to have similar molecule structures as medical triazoles showed significantly higher MICs for multi triazole resistant TR_34_/L98H *A. fumigatus* from environmental and clinical samples than those of wild type strains (Table 2). In addition, metconazole and hexaconazole also showed high MICs for multi-triazole resistant *A. fumigatus* isolates with the TR_34_/L98H mutation. Attention is called to the report of Serfling et al., who used the maize anthracnose fungus *Colletotrichum graminicola* model system to study the acquisition of azole resistance and investigated whether isolates that were resistant to an agricultural azole show cross-resistance to azoles and antifungal agents of other chemical classes used in medicine [30]. Their in-vitro data revealed that *C. graminicola* was able to efficiently adapt to medium containing azoles, and strains adapted to tebuconazole were less sensitive to all agricultural and medical azoles tested than the non-adapted control strain. Likewise, azole cross-resistance was observed for yeast isolates from the oropharynx of human immunodeficiency virus-infected patients to agricultural azole drugs and for those from environmental sources to medical azole drugs [31].

It is remarkable that all of the environmental and clinical TR_34_/L98H *A. fumigatus* isolates in India had the same microsatellite genotype. Although the environmental isolates originated from geographically diverse regions of northern, eastern and southern parts of India were separated from each other by about 2000 Km, they harboured an identical short tandem repeat (STR) pattern. The possibility of contamination during handling of samples was ruled out by processing of the samples by different laboratory personnel in two different laboratories in India and the Netherlands. Furthermore, we had reported earlier that two clinical TR_34_/L98H *A. fumigatus* isolates originating from two azole naive patients, who were residents of Bihar and Delhi, shared the same STR pattern [22]. Moreover, azole-resistant strains from the environment of Bihar and Delhi also showed the same STR pattern. Notably, genetic analysis of a collection of MTR isolates showed that all isolates with the TR_34_/L98H allele were all confined within a single clade and were less variable than susceptible isolates [25], consistent with a single and recent origin of the resistant genotype.

Our results are consistent with the hypothesis that the azole-resistant *A. fumigatus* strains analyzed here from across India were due to the clonal spread of a single genotype. The lack of a single azole-susceptible strain from either clinical origin or the environment in India with the same genotype as the widespread azole-resistant genotype it may be conceivable that the resistant genotype was unlikely the result of a single mutation at the *cyp*51A gene in a common azole-susceptible genotype in India. In addition, our genotype analysis suggest that the azole-resistant genotype in India was likely an extremely adaptive recombinant progeny derived from a cross between an azole-resistant strain migrated from outside of India and a native azole-susceptible strain from within India, followed by mutation. The abundant phylogenetic incompatibility found in each of the sub-samples as well as in the whole sample (where 100% of the loci pairs were phylogenetically incompatible, thus consistent with recombination) supports sexual mating in natural populations of this species in India. Our inferred mechanisms have been similarly suggested for the emergence of many virulent strains of viral, bacterial and protozoan pathogens [32], [33]. Once the extremely fit *A. fumigatus* genotype emerged in India, it could spread quickly by producing a large number of airborne asexual spores in the environment. These airborne spores can easily disperse to other geographic areas by air current or anthropogenic means. The widespread application of triazole fungicides in the environment in India in the last two decades could have contributed to its spread by reducing the azole-susceptible genotypes and selecting for this azole-resistant genotype. Whether this resistant genotype has spread to neighbouring countries remain to be determined.

## Materials and Methods

### Ethics Statement

All necessary permits were obtained for the described field studies.

### Collection of Environmental Samples

A total of 486 environmental samples including soil from flowerbeds of nurseries, surrounding parks of hospitals, cotton trees, tea gardens, paddy fields, soil containing bird excreta, decayed wood of tree trunks and aerial samples of the indoor environment of hospital wards from the Union Territory (UT) of Delhi, Haryana, Himachal Pradesh, Uttrakhand, Bihar, West Bengal, Sikkim, Meghalaya and Tamil Nadu States were investigated during July 2011–April 2012. The distribution of the investigated 486 samples was as follows: UT of Delhi (n = 266), Haryana (n = 21), Himachal Pradesh (n = 4), Uttrakhand (n = 21), Bihar (n = 33), West Bengal (n = 59), Sikkim (n = 6), Meghalaya (n = 11) and Tamil Nadu (n = 65).

### Soil and Aerial Sampling

About two gram of soil was suspended in 8 ml of 0.85% NaCl, vortexed and allowed to settle for 30 seconds. Subsequently, the suspension was diluted 1∶10 and 100 µl was plated in duplicates on Sabouraud dextrose agar plates supplemented with 50 mg/L chloramphenicol and incubated at 37°C for 48 h. One gram of decayed wood was suspended in 10 ml of 0.85% NaCl and allowed to settle after vortexing it for 1 min. Then, 100 µl of suspension was plated in duplicates on SDA and incubated at 37°C for 48 h.

For the indoor aerial sampling of the hospital, duplicate SDA plates were exposed for 1 h in the corners and centre of the general outpatient and wards of the V. P. Chest Institute (VPCI), Delhi, on two different occasions. Plates were incubated for 48 h at 37°C.

### Identification

In order to detect overall prevalence of *A. fumigatus* the samples were initially inoculated on SDA plates and maximum of 3 colonies per plate were purified and identified by macro- and microscopic characteristics and growth at 50°C which differentiated *A. fumigatus* from *A. lentulus*. Samples found out to be negative for *A. fumigatus* were again processed without dilution and inoculated directly on SDA plates. All of the *A. fumigatus* isolates were then subcultured on SDA plates supplemented with 4 mg/L itraconazole and incubated at 37°C for 48 h. Identification of all the *A. fumigatus* isolates that grew on 4 mg/L itraconazole containing SDA plates (ITC+ isolates) were confirmed by sequencing of the internal transcribed spacer region. In order to rule out any cryptic species within *Aspergillus* section Fumigati, molecular identification was performed by amplification of parts of the β-tubulin gene and calmodulin gene [34], [35].

### Antifungal Susceptibility Testing

The in vitro activity of all the standard azole antifungals was investigated using CLSI M38-A2 broth microdilution [36]. A total of 53 itraconazole resistant *A. fumigatus* isolates (44 ITC+ environmental and 9 ITC+ clinical) were subjected to AFST. Nine itraconazole resistant clinical isolates were cultured from patients suspected of bronchopulmonary aspergillosis. Among the 9 ITC+ *A. fumigatus* clinical isolates two have been reported earlier [22]. In addition, 35 itraconazole susceptible *A. fumigatus* isolates comprising 22 randomly selected wild type environmental and 13 azole susceptible clinical *A. fumigatus* isolates cultured from patients of suspected bronchopulmonary aspergillosis were included as controls. The drugs tested included itraconazole (ITC, Lee Pharma, Hyderabad, India, and Janssen Research Foundation, Beerse, Belgium), voriconazole (VRC, Pfizer Central Research, Sandwich, Kent, United Kingdom) and posaconazole (POS, Schering-Plough, Kenilworth, NJ, USA, now Astellas). For the broth microdilution test, RPMI 1640 medium with glutamine without bicarbonate (Sigma-Aldrich, St Louis, MO, USA) buffered to pH 7 with 0.165 M 3-N-morpholinepropanesulfonic acid (Sigma) was used. Isolates were grown on potato dextrose agar for 5 days at 28°C and the inoculum was adjusted to a final density of 0.5–2.5 x 10^4^ cfu/ml by measuring 0.09–0.13 OD at 540 nm using spectrophotometer. The final concentrations of the drugs were 0.03 to 16 mg/L for itraconazole and voriconazole and 0.015 to 8 mg/L for posaconazole. Drug-free and mould-free controls were included and microtitre plates were incubated at 35°C for 48 h. CLSI recommended quality control strains, *Candida krusei,* ATCC6258 and *Candida parapsilosis,* ATCC22019 and reference strains *Aspergillus fumigatus,* ATCC204305 and *Aspergillus flavus,* ATCC204304 were included. The MIC end points were read visually which, for azoles were defined as the lowest concentration at which there was 100% inhibition of growth compared with the drug-free control wells. *A. fumigatus* isolates with high itraconazole MICs were tested twice on different days. Azole resistance was defined for itraconazole, >2 mg/L, voriconazole, >2 mg/L, and posaconazole, >0.5 mg/L as proposed by Verweij et al. [37].

### Activity of Azole Fungicides

The commonly used ten azole fungicides registered under the Insecticides Act, 1968 by the Indian Central Insecticide Board and Registration Committee were tested for activity against resistant and wild type environmental and clinical *A. fumigatus* Indian isolates by microdilution method as described above. The azole fungicides tested were bromuconazole, cyproconazole, difenoconazole, epoxiconazole, penconazole, tebuconazole, triadimefon, metconazole (kindly gifted by Dr. P. Verweij, Nijmegen, the Netherlands) hexaconazole (Rallis India, Mumbai, India) and tricyclazole (Cheminova India, Mumbai, India). The fungicides were dissolved in dimethyl sulfoxide and concentration range used was 0.06–32 mg/L.

### Statistical Analysis

Point serial correlation was computed between MICs of wild type and TR_34_/L98H *A. fumigatus* isolates of clinical and environmental origin to determine the correlation coefficient which is a measure of the effect size (r), where values of r = 0 indicate no correlation between MICs, r = 1 indicate positive correlation and r = −1 indicate negative correlation. In cases where correlation MICs have similar values for all isolates, correlation effect size was considered r = 0 [21].

### Mixed Format Real-time PCR Assay to Detect Mutations

All of the ITC+ *A. fumigatus* isolates were subjected to a mixed-format real-time PCR assay as described previously for detection of TR_34_/L98H, TR_46_/Y121F/T289A, M220, G54 mutations leading to triazole resistance in *A. fumigatus* [38].

### Microsatellite Genotypic Analysis

Genotyping was performed with a panel of nine short tandem repeats as described previously [39]. The genetic relatedness between Indian environmental and clinical isolates was determined by using microsatellite typing. A total of 60 ITC+ *A. fumigatus* isolates which included 51 environmental (44 isolated in the Indian laboratory and 7 isolated from Indian soil samples processed in the Netherlands laboratory) and 9 clinical isolates were subjected to microsatellite typing. For phylogenetic analysis, 24 Dutch (15 clinical and 9 environmental), 8 clinical Chinese [40], 3 clinical French [18] and one clinical German [19] isolates of *A. fumigatus* containing the TR_34_/L98H genotype were tested along with the Indian isolates. In addition, 35 (22 environmental and 13 clinical) Indian, 12 environmental Dutch and 2 clinical French *A. fumigatus* isolates without mutations and a reference strain *A. fumigatus* AF293 were included in the analysis.

### Genetic Analysis of Microsatellite Genotypes

The composite genotype for each of the 146 strains of *A. fumigatus* was identified based on alleles at all nine microsatellite loci. The genotype information was then used to identify genetic relationships among strains. Gene diversity and genotype diversity within individual samples and the relationships between samples were estimated using the population genetic analyses program GenAlEx 6.1 [41]. The relationships among alleles at different loci were examined for evidence of recombination in natural populations of this fungus, using the computer program Multilocus 2.0 (http://www.agapow.net/software/multilocus/) [42]. Results of these analyses were used to infer the potential source(s) of the triazole-resistant clinical and environmental *A. fumigatus* strains in India.
